# Preoperative imaging markers and PDZ-binding kinase tissue expression predict low-risk disease in endometrial hyperplasias and low grade cancers

**DOI:** 10.18632/oncotarget.19708

**Published:** 2017-07-31

**Authors:** Anna Berg, Ankush Gulati, Sigmund Ytre-Hauge, Kristine E. Fasmer, Karen K. Mauland, Erling A. Hoivik, Jenny A. Husby, Ingvild L. Tangen, Jone Trovik, Mari K. Halle, Ingunn Stefansson, Lars A. Akslen, Kathrine Woie, Line Bjørge, Helga B. Salvesen, Øyvind O. Salvesen, Henrica M.J. Werner, Ingfrid S. Haldorsen, Camilla Krakstad

**Affiliations:** ^1^ Centre for Cancer Biomarkers, Department of Clinical Science, University of Bergen, Bergen, Norway; ^2^ Department of Gynecology and Obstetrics, Haukeland University Hospital, Bergen, Norway; ^3^ Department of Radiology, Haukeland University Hospital, Bergen, Norway; ^4^ Section of Radiology, Department of Clinical Medicine, University of Bergen, Norway; ^5^ Department of Pathology, Haukeland University Hospital, Bergen, Norway; ^6^ Department of Clinical Medicine, Centre for Cancer Biomarkers, Bergen, Norway; ^7^ Unit for Applied Clinical Research, Department of Cancer Research and Molecular Medicine, Norwegian University of Science and Technology, Trondheim, Norway

**Keywords:** endometrial carcinoma, endometrial hyperplasia, PBK, MRI, FDG-PET/CT

## Abstract

**Purpose:**

Distinguishing complex atypical hyperplasia (CAH) from grade 1 endometrioid endometrial cancer (EECG1) preoperatively may be valuable in order to prevent surgical overtreatment, particularly in patients wishing preserved fertility or in patients carrying increased risk of perioperative complications.

**Material and methods:**

Preoperative histological diagnosis and radiological findings were compared to final histological diagnosis in patients diagnosed with CAH and EECG1. Imaging characteristics at preoperative magnetic resonance imaging (MRI) and fluorodeoxyglucose positron emission tomography/computer tomography (FDG-PET/CT) were compared with tumor DNA oligonucleotide microarray data, immunohistochemistry findings and clinicopathological annotations.

**Results:**

MRI assessed tumor volume was higher in EECG1 than in CAH (p=0.004) whereas tumor apparent diffusion coefficient value was lower in EECG1 (p=0.005). EECG1 exhibited increased metabolism with higher maximum and mean standard uptake values (SUV) than CAH (p≤0.002). Unsupervised clustering of EECG1 and CAH revealed differentially expressed genes within the clusters, and identified PDZ-binding kinase (PBK) as a potential marker for selecting endometrial lesions with less aggressive biological behavior.

**Conclusion:**

Both PBK expression and preoperative imaging yield promising biomarkers that may aid in the differentiation between EECG1 and CAH preoperatively, and these markers should be further explored in larger patient series.

## INTRODUCTION

Pathological proliferation of the endometrium ranges from mild and reversible glandular proliferation, to premalignant lesions [1]. In patients initially diagnosed with endometrial hyperplasia based on preoperative biopsy, approximately 20% will have a change of diagnosis to endometrial cancer when based on histological assessment of the hysterectomy specimen [2]. For patients with endometrial hyperplasia with cellular atypia, i.e. complex atypical hyperplasia (CAH) and atypical hyperplasia (AH) the proportion of patients diagnosed with concomitant endometrial cancer after hysterectomy is 43% [3]. Therefore, patients diagnosed with premalignant endometrial lesions are usually recommended a total hysterectomy [4]. However, in long-term follow-up studies (13-20 years) approximately 70% of the CAHs, regarded as the most severe form of premalignant endometrial lesions, do not progress to cancer [5, 6]. Thus, significant overtreatment of patients with premalignant endometrial lesions seems to exist. The reported limitations in accurately differentiating between the different subtypes of endometrial hyperplasia and between hyperplasia and malignant lesions from endometrial biopsies, call for biomarkers to support treatment decisions [7].

Improved diagnostic methods to aid differentiation between true endometrial hyperplasias and endometrial cancer preoperatively could be beneficial for several reasons. For women preferring fertility sparing treatment, or in patients with severe co-morbidity or high body mass index (BMI), carrying an increased risk of surgical complications, conservative hormonal treatment may represent a favorable alternative [8–10]. This would also reduce the cost related to unnecessary surgical treatment of patients with premalignant endometrial lesions [11, 12].

Preoperative imaging by magnetic resonance imaging (MRI) and fluorodeoxyglucose positron emission tomography/computer tomography (FDG-PET/CT) has been shown to be useful in the preoperative staging of endometrial cancer and for identifying high risk endometrial cancer patients [13, 14]. However, the value of preoperative MRI and FDG-PET/CT to differentiate premalignant endometrial lesions from low risk endometrial cancer is not known [13, 15].

This study explores the molecular alterations characteristic of low grade lesions, aiming to identify molecular markers that may aid in the discrimination between complex atypical hyperplasias and grade 1 endometrioid endometrial cancers (EECG1). Furthermore, we explore the corresponding preoperative morphological and functional imaging findings of these lesions, to identify potential imaging biomarkers that could aid in developing personalized treatment algorithms.

## RESULTS

### Preoperative imaging parameters differentiate between CAH and EECG1

The final diagnosis based on the hysterectomy specimen was CAH in 46% (110/238) and EECG1 in 54% (128/238) of the patients (Table 1). The preoperative endometrial biopsy suggested CAH in 31% (74/238), EECG1 in 40% (96/238), and was inconclusive in 29% (68/238) (Table 1). Only 4% (3/74) of the patients categorized as CAH based on preoperative biopsy were diagnosed as EECG1 at hysterectomy, whereas among patients with inconclusive preoperative biopsy, 43% (29/68) had a final diagnosis of EECG1. All patients defined as EECG1 based on preoperative biopsy were confirmed as EECG1 after hysterectomy (Table 1).

**Table 1:** Preoperative diagnosis based on endometrial biopsy, pelvic MRI and FDG-PET/CT, and corresponding final histological diagnosis at hysterectomy in patients with CAH and EECG1

Postoperative diagnosis from hysterectomy specimen			
**Preoperative diagnosis from endometrial biopsy, n (%) n=238**	**CAH (n=110) n (%)**	**EECG1 (n=128) n (%)**	**p-value^a^**
CAH, n=74 (31)	71 (96)	3 (4)	**<0.001**
Inconclusive, n=68 (29)	39 (57)	29 (43)	
EECG1, n= 96 (40)	0 (0)	96 (100)	
**MRI finding n=117**	**CAH (n=23) n (%)**	**EECG1 (n=94) n (%)**	**p-value^b^**
No or barely visible tumor	9 (39)	0 (0)	**<0.001**
Visible tumor	14 (61)	94 (100)	
Myometrial invasion			
No or <50%	23 (100)	63 (67)	**0.001**
>50%	0 (0)	31 (33)	
Cervical stroma infiltration			
No	23 (100)	88 (94)	0.60
Yes	0 (0)	6 (6)	
Enlarged pelvic lymph nodes			
No	21 (91)	92 (98)	1.0
Yes	2(9)^c^	2 (2)	
**FDG-PET/CT finding n=69**	**CAH (n=10) n (%)**	**EECG1 (n=59) n (%)**	**p-value^b^**
No visible tumor	4 (40)	1 (2)	**0.001**
Increased tumor avidity	6 (60)	58 (98)	
Cervical FDG-avidity			
No	10 (100)	48 (81)	0.40
Yes	0 (0)	11 (19)	
Enlarged FDG avid lymph nodes			
No	9 (90)	56 (95)	0.50
Yes	1 (10)^d^	3 (50)	

^a^Pearson Chi-Square test. ^b^Fisher's Exact Test. ^c^Two patients with lymphadenopathy in the pelvis and groin due to lymphoma. ^d^One patient with enlarged lymph nodes due to lymphoma. Significant p-values are given in **bold**.

**Figure 1:**
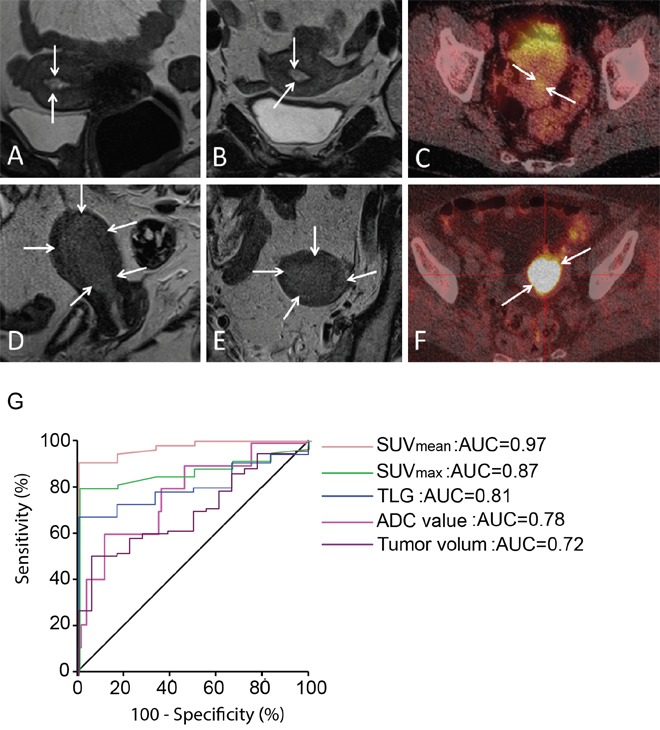
Preoperative imaging in premalignant and malignant endometrial lesions **(A-B)** Sagittal (A) and axial (B) T2-weighted MRI depicting a small uterine lesion (arrows) with volume of 0.5 cm^3^ in CAH patient. **(C)** FDG-PET/CT in the same patient demonstrates that the lesion (arrows) exhibits low FDG avidity (with SUV_mean_ of 1.7 g/ml). **(D-E)** Sagittal (D) and axial (E) T2-weighted MRI depicting a large uterine tumor (arrows) with volume of 18.7 cm^3^ in a patient with EECG1. **(F)** FDG-PET/CT in the same patient shows that the lesion (arrows) exhibits high FDG avidity (with SUV_mean_ of 11.1 g/ml). **(G)** ROC curves for the different imaging parameters (PET/CT: SUV_mean_, SUV_max_ and TLG. MRI: ADC value and tumor volume) for the discrimination of EECG1 from CAH shows that SUV_mean_ yielded the best area under curve (AUC=0.97).

At preoperative MRI a detectable endometrial tumor was observed in 61% (14/23) of patients with final diagnosis of CAH as opposed to 100% (94/94) of patients with EECG1 (p<0.001) (Table 1). None of the CAHs had signs of deep myometrial invasion, whereas MRI depicted tumor invading >50% of the myometrial wall in 33% (31/94) of EECG1 patients (p=0.001). Increased FDG avidity of the endometrial lesion was observed in 60% (6/10) of the CAHs as opposed to 98% (58/59) of the EECG1 lesions (p=0.001) (Table 1). The frequency of imaging findings suggestive of cervical tumor infiltration or lymph node metastases was rare, both at MRI and PET, and was not significantly different for CAH and EECG1 (Table 1). Quantitative imaging parameters derived from visible endometrial lesions showed that CAH exhibited significantly lower lesion volume than EECG1 (with mean volume of 2.3 ml versus 11.7 ml, respectively; p=0.004) and exhibited higher lesion ADC value (p= 0.005) (Table 2) (Figure 1). Additionally, lesion maximum standardized uptake value (SUV_max_), mean standardized uptake value (SUV_mean_) and total lesion glycolysis (TLG) were all significantly lower in patients having final diagnosis of CAH compared to EECG1 (p≤0.01 for all) (Table 2) (Figure 1).

**Table 2:** Quantitative imaging parameters from endometrial lesions at preoperative MRI and FDG-PET/CT in 110 patients with CAH and EECG1

Quantitative imaging parameter	Histological type		p-value*
	CAH Mean (n§)	EECG1 Mean (n§)	
**MRI**			
Tumor ADC value (× 10^−6^ mm^2^/s)	1071 (10)	851 (88)	**0.005**
Tumor volume (ml)	2.3 (18)	11.7 (92)	**0.004**
**FDG PET-CT**			
Tumor SUV_max_	4.6 (6)	12.6 (58)	**0.002**
Tumor SUV_mean_	2.7 (6)	5.3 (55)	**<0.001**
MTV (ml)	8.2 (6)	23.1 (55)	0.1
TLG (g)	20.8 (6)	154.5 (55)	**0.01**

* Mann Whitney U test. § n refers to the number of cases eligible for calculation of quantitative imaging parameters (visible or barely visible endometrial lesion being a prerequisite). Significant p-values are given in **bold**.

Receiver operating characteristic (ROC) curves were generated for the imaging parameters (apparent diffusion coefficient (ADC) value, tumor volume, SUV_max_, SUV_mean_ and TLG) that were significantly different in EECG1 and CAH (Figure 1G). For the discrimination between EECG1 and CAH, lesion SUV_mean_ had the highest area under curve (AUC=0.97; p<0.001), and the derived cut-off value of SUV_mean_ > 3.2 yielded a sensitivity of 89 % (49/55) and a specificity of 100% (6/6) for the prediction of EECG1.

### Gene clusters suggest specific pathogenic drivers in early endometrial carcinogenesis

**Figure 2:**
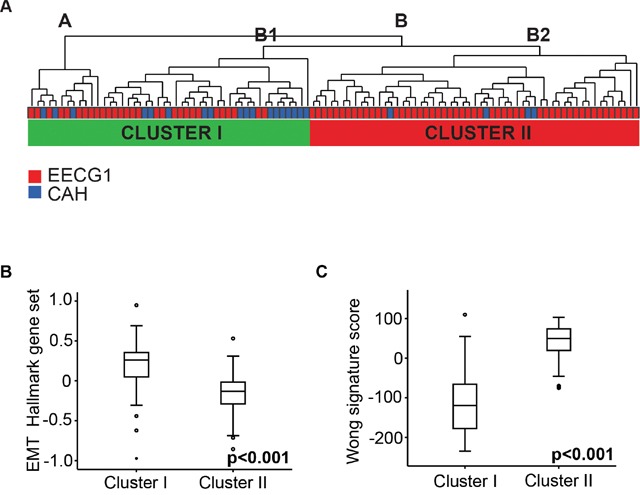
Gene expression analysis of lesions with premalignant and low grade endometrioid endometrial cancer **(A)** Unsupervised clustering of 102 patients with CAH and EECG1 including all genes available in applied array. **(B)** EMT Hallmark gene sets in Cluster I and II. **(C)** Wong signature score in Cluster I and II.

Supervised gene expression analysis by significance analysis of microarrays (SAM) revealed no significantly differentially expressed genes between lesions classified as CAH or EECG1 by final histological diagnosis. We therefore pooled premalignant and low grade malignant endometrial lesions together and performed an unsupervised clustering of this cohort (n=102) (Figure 2A). This analysis yielded two main clusters (A and B), with cluster B divided into two sub-clusters, B1 and B2. Cluster B1 was histologically more similar to cluster A than to cluster B2. Cluster A and B1 also showed closest proximity in Correspondence analysis [16] (data not shown). Based on this we separated the patients in two clinical clusters, Cluster I and Cluster II (Figure 2A). Interestingly, 82% (18/22) of patients with CAH were found in Cluster I. There were no significant differences in age at hysterectomy, BMI, parity or menopausal status between Cluster I and II.

To explore biological differences between Cluster I and Cluster II, gene set enrichment analysis (GSEA) was performed applying gene sets from Molecular Signatures Database (MSigDB, version 5.0) [17]. Top-ranked Hallmark gene sets and curated gene sets significantly up-regulated in Cluster I and in Cluster II are shown in Supplementary Table 1 and 2. The epithelial to mesenchymal transition (EMT) Hallmark gene set was the most significantly up-regulated gene set in Cluster I (Figure 2B). When applying curated gene sets (C2), a gene set representing gene alterations in endometrioid endometrial cancer compared to normal endometrial tissue was top-ranked (WONG_ENDOMETRIUM_DN up-regulated in Cluster I) [18] (Supplementary Table 2). We calculated a gene signature score based on the total gene set, (WONG_ENDOMETRIUM_CANCER_DN and WONG_ENDOMETRIUM_CANCER_UP), by subtracting the down-regulated genes from the up-regulated as previously described [19]. We found this signature score to be highly significantly increased in lesions in Cluster II (Figure 2C).

### PBK predicts an aggressive phenotype in low grade endometrial lesions

The 25 top-ranked genes separating the two clusters are listed in Supplementary Table 3. The four genes, PDZ-binding kinase (*PBK*), never in mitosis related kinase 2 (*NEK2*), maternal embryonic leucine zipper kinase (*MELK*) and cyclin B1 (*CCNB1*), separated the clusters most accurately based on the narrowest and non-overlapping confidence intervals when comparing the two clusters. Among these genes, *PBK* and *NEK2* have previously been suggested as biomarkers for aggressive disease in several types of cancer [20–25]. Preliminary analysis of PBK and NEK2 protein expression were conducted in a small subset of patients (n=55). NEK2 was not found to be a predictive marker for early invasive endometrial cancer in our data set, possibly due to unspecific binding of the applied antibody. On the other hand, PBK demonstrated promising results. Subsequently, immunohistochemistry (IHC) was conducted for PBK protein expression in the total patient cohort, and results analyzed in relevant subgroups.

**Figure 3:**
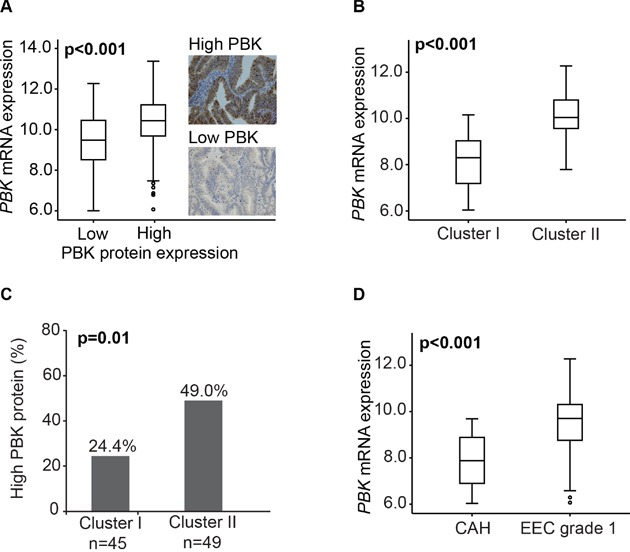
PBK mRNA and protein expression in premalignant and low grade endometrioid endometrial cancer lesions **(A)** *PBK* mRNA expressions level in association with PBK protein expression in 205 patients with overlapping data. **(B)** *PBK* mRNA expression in Cluster I and Cluster II in the 102 included patients. **(C)** PBK protein expression in Cluster I and Cluster II in 94 patients with overlapping data. **(D)** *PBK* mRNA expression associated with final histological diagnosis CAH or EECG1 in the 102 patients included.

PBK protein expression was highly significantly associated to *PBK* mRNA expression (p<0.001) (Figure 3A). The good separation of Cluster I and II by PBK mRNA expression (Figure 3B) was validated in protein expression analysis finding PBK protein expression to be significantly higher in Cluster II than Cluster I (Figure 3C). Also, *PBK* mRNA level was significantly higher in EECG1 than CAH lesions (Figure 3D).

In line with this, we found PBK protein expression to be associated to deep myometrial infiltration, lymph node metastasis and loss of progesterone receptor (PR) (Table 3). Also supporting PBK as a marker of aggressive biology, we found high PBK protein expression to be associated with reduced disease specific survival in endometrioid endometrial cancer (n=410; p=0.05) (Supplementary Figure 1A). Furthermore, both the EMT Hallmark gene sets and the Wong signature score were significantly correlated to *PBK* mRNA expression level (with rs=-0.35, p<0.001 and rs=0.56, p<0.001, respectively) (Supplementary Figure 1B-1C).

**Table 3:** Correlation between PBK protein expression and clinicopathological characteristics in patients with CAH (histological type and grade, ERα and PR protein expression) and endometrioid endometrial cancer (EEC) (additionally myometrial infiltration, lymph node metastasis and FIGO stage)

Variable	Categories	PBK protein expression		
		Low n (%)	High n (%)	p-value*
Histological type & grade	CAH	64 (84)	12 (16)	**<0.001**
n=486	EEC Grade 1	141 (70)	60 (30)	
	EEC Grade 2	81 (59)	56 (41)	
	EEC Grade 3	29 (40)	43 (60)	
Myometrial infiltration	< 50%	166 (65)	88 (35)	**0.03**
n=409	> 50%	85 (55)	70 (45)	
Lymph node metastasis	No	187 (62)	116(38)	**0.006**
n=327	Yes	8 (33)	16 (67)	
ERα protein expression	High	262 (66)	138 (34)	0.60
n=480	Low	48 (60)	32 (40)	
PR protein expression	High	287 (67)	143 (33)	**0.005**
n=481	Low	24 (47)	27 (53)	

* Chi-square test. n, number of patients assessed for PKB protein expression. Significant p-values are given in **bold**.

We included CAH and endometrioid endometrial cancer with the lowest histological grade in our hypothesis-generating approach. However, 56 patients with available PBK expression data and 27 patients with mRNA data, and EECG1, had international federation of gynecology and obstetrics (FIGO) stage over IA (mainly deep myometrial infiltration, FIGO IB) after hysterectomy. In sub-analysis including this knowledge, we found the significant increase in *PBK* mRNA to occur from CAH to EECG1 stage IA (Supplementary Figure 2A, p≤0.001). This is also true for PBK protein expression, being significantly higher in EECG1 stage IA than CAH (p=0.04) (Supplementary Figure 2B). However, there is no significant increase in PBK mRNA or protein expression from EECG1 stage IA to stage IB or more (Supplementary Figure 2A-2B). This indicates that the clustering is not driven by the more invasive lesions included in this analysis.

### Imaging parameters are associated with the gene clusters

In patients with available imaging data overlapping with the gene expression array, we found tumor ADC value to be highly significantly lower in lesions found in Cluster II (Supplementary Table 4). We also observed significantly larger tumor volume and increased tumor metabolism (SUV_max_, SUV_mean_ and TLG) in lesions in Cluster II (p≤0.03 for all; Supplementary Table 4). We also investigated whether PBK expression was associated with the same imaging parameters. Tumor volume, SUV_max_ and SUV_mean_ were significantly higher in lesions with high PBK protein expression (p≤0.03 for all; Supplementary Table 5) and *PBK* mRNA expression was significantly anti-correlated to ADC value in low grade endometrial cancer and CAH (p=0.03, data not shown).

## DISCUSSION

In this study we have comprehensively assessed a large patient series with CAH and EECG1, and identified a promising prognostic immunohistochemical marker, putatively reflecting important aspects related to early endometrial carcinogenesis. This study also suggests a very promising role of diagnostic imaging by MRI and FDG-PET/CT yielding imaging biomarkers that may aid in the preoperative differentiation between CAH and EECG1. These tissue and imaging biomarkers should be further studied as candidate biomarkers for inclusion in risk-stratified treatment algorithms, and may aid in identifying low-risk patients who may be eligible for non-surgical treatment strategies.

We found that almost 30% of patients with final diagnosis of CAH and EECG1 had an inconclusive/uncertain preoperative diagnosis. This probably reflects the challenges in the diagnostics of these patients. The reproducibility in diagnosing atypical hyperplasia (AH) has been demonstrated to be low, even amongst expert pathologists [7]. The poor reproducibility of the pathological diagnosis and a near 50% risk of concurrent carcinoma in the hysterectomy specimen are the main reasons why premalignant disorders are normally treated with hysterectomy [3, 7, 26]. In this study, only 4% of patients classified as CAH preoperative had EECG1 after hysterectomy. However, in a large portion of preoperative samples the diagnosis was uncertain, and probably more patients would have discordant diagnosis prior to and after hysterectomy if the preoperative diagnosis had been more conclusive. The classification system of endometrial intraepithelial neoplasia (EIN) is reportedly superior to the WHO system in predicting progression to cancer, and has been shown to improve the diagnostic reproducibility for the diagnosis of EIN [12]. Accordingly, the EIN classification system is now implemented in new the WHO classification guidelines [27]. However, in both systems the diagnosis of atypia is hampered by subjectivity, and this illustrates the limitations of routine histopathological diagnosis. Over the past decade, there have been suggested molecular biomarkers to predict the risk of carcinoma in patients preoperatively diagnosed as endometrial hyperplasia, including loss of estrogen receptor (ER), PR and phosphatase and tensin homolog (PTEN) protein expression [28, 29]. However, no biomarkers are yet routinely implemented in the clinic for this use. Identification of endometrial cancer with an expected less aggressive nature can also be valuable since the risk associated with surgical treatment of patients with co-morbidities and low- to medium risk endometrial cancer sometimes exceed the predicted survival benefits [30]. Biomarker to improve distinction between premalignant and malignant endometrial lesions could potentially select patients for conservative regime in patients were surgical treatment is not preferred.

Routinely performed preoperative MRI and/or FDG-PET/CT showed that ∼60% of patients with final diagnosis of CAH had MRI and FDG-PET/CT findings suggesting presence of an endometrial tumor. Furthermore, no or barely visible tumor at preoperative MRI and/or PET/CT was almost exclusively observed in CAH and a very small lesion size with low metabolic activity was highly suggestive of CAH. Interestingly, we found that employing a cut-off value for tumor SUV_mean_ >3.2, yielded a sensitivity of 89% and a specificity of 100% for diagnosing EECG1. Similarly, SUV_mean_ ≤ 3.2 yielded a sensitivity of 100% and a specificity of 89% for the diagnosis of CAH. Thus, lesion SUV_mean_ is a very promising biomarker that may aid in identifying patients that are very likely to have CAH or low-risk disease. We acknowledge that most centers do not routinely perform preoperative FDG-PET/CT in patients with suspected premalignant and low grade malignant endometrial lesions, however, in selected patients in whom non-surgical treatment could be preferable, PET/CT may provide information supporting a diagnosis of CAH.

Unsupervised analysis of gene expression data revealed *PBK* as an important gene separating the CAH and EECG1 lesions by genetic variances. This approach was chosen based on low diagnostic reproducibility of the pathological diagnosis of CAH [7], and the apparent lack of molecular differences in our cohort when comparing CAH to EECG1. PBK has been demonstrated to be linked to aggressive phenotype in prostate-, breast- and cervical cancer [20, 22, 31]. Importantly, PBK has been shown to be central for the invasive and migratory function of prostate cancer cells [31]. Also, in gastric cancer cell line studies, migration and invasion were reduced by knockdown of *PBK* [21]. The same authors demonstrated in a patient series (n=48) with gastric adenocarcinoma, that high nuclear PBK protein expression was associated with an invasive phenotype, higher stage, deep invasion and lymph node metastasis [21]. Interestingly, in cervical intraepithelial neoplasia (CIN), PBK protein expression has been demonstrated to incrementally increase in higher grade premalignant lesions and further to cancer and increasing PBK protein expression level is linked to more aggressive tumors [22]. Consistent with these findings our results indicate that PBK protein and mRNA expression have potential as a selection marker in premalignant and low grade malignant endometrial lesions. We also demonstrate that this biomarker is associated with imaging parameters, and that therefore similar information may be obtained non-invasively from routinely used diagnostic imaging methods. Importantly, an identified PBK inhibitor, HI-TOPK-032, has been shown to inhibit tumor growth, both *in vitro* and *in vivo*, making this biomarker interesting for further clinical studies [32, 33].

In conclusion, this comprehensive study of premalignant and low grade malignant endometrial cancer lesions suggests that PBK may represent a novel immunohistochemical marker reflecting biological mechanisms important for endometrial carcinogenesis that may be potentially targetable by novel drugs. Furthermore, preoperative imaging by MRI and PET/CT yields imaging markers that are closely related to tumor PBK expression levels, and these imaging markers may potentially aid in the discrimination between CAH and endometrial cancer. A combination of tissue biomarkers and imaging biomarkers may potentially improve the preoperative risk-stratification of endometrial lesions, and should be further explored as candidate biomarkers to identify patients with likely CAH in whom non-surgical treatment may represent a safe alternative. Validation of these markers in larger and randomized prospective studies is needed prior to implementation in the clinic.

## MATERIALS AND METHODS

### Patient and tissue samples

All surgically treated patients with a postoperative diagnosis of CAH or EECG1 (all FIGO stages) treated at Haukeland University Hospital between May 2001 and January 2015 were included in the study. All patients consented to the collection of imaging data and tissue specimens for biomarker studies as part of institutional review board-approved protocols (Rek Vest 2009/2315; 2015/2333).

Tumor tissue was sampled from the hysterectomy specimens and stored in the Bergen Gynecologic Cancer Biobank. Clinical and histopathological data were recorded from the medical records. In total, 277 patients with primary endometrioid endometrial cancer grade 1 (n=201) and CAH (n=76) were included. A subset of 80 EECG1 and 22 CAH patients also had fresh frozen tissue, used for RNA extraction and gene expression analysis. Patients with endometrial cancer or CAH have been routinely subjected to preoperative diagnostic imaging by contrast-enhanced (CE)-MRI (since 2009) and FDG-PET/CT (since 2011) at our institution. In this study, 117 patients (EECG1=94 and CAH=23) and 69 patients (EECG1=59 and CAH=10) had undergone preoperative CE-MRI and FDG-PET/CT, respectively. For EECG1 patients with imaging data and/or gene expression data, and all included CAH patients, data from routine pathology evaluation was used. Inconclusive preoperative diagnosis was defined as “CAH, could not rule-out cancer” or “CAH, with possible invasive cancer”. For patients with final diagnosis EECG1 in which preoperative pathology report was revisited (n=128) we did not identify lesions with grade 2-3 in preoperative samples.

Clinical information included age at hysterectomy, BMI, menopausal status, parity, primary surgical treatment and adjuvant therapy received. For patients with EECG1, FIGO stage, histologic subtype, grade and follow-up data were also recorded. For evaluation of identified tissue biomarkers, a larger cohort of EEC (n=410, grade 1, 2 and 3) was included, in addition to 76 CAH patients. This cohort is previously described [34].

### Magnetic resonance imaging protocol and derived imaging parameters

Preoperative MRI was conducted on a whole-body 1.5-T MRI system (Siemens Avanto running Syngo v. B17) using a six-channel body coil applying a standardized imaging protocol [35]. To reduce motion artefacts 20 mg butylscopolamine bromide (Buscopan; Boehringer, Ingelheim) was administered intravenously just prior to scanning. Mean/median (range) interval between MRI examination and surgery was 17/12 (0–175) days. Structural MRI included pelvic sagittal and axial oblique (perpendicular to the long axis of the uterus) T2-weighted images and axial oblique T1-weighted gradient-echo images. T1-weighted series were acquired before and after intravenous administration of gadoterate meglumine (Dotarem, Guerbet: 0.1 mmol gadolinium per kilogram of body weight, 3 ml/s injection speed) using a 2- min delay. Diffusion weighted imaging (DWI) of the pelvis was acquired using an axial two-dimensional echo planar imaging (EPI) sequence with b-values of 0 and 1000 s/mm^2^ with calculation of ADC maps. Lymph nodes were considered enlarged when the short-axis diameter exceeded 10 mm [36]. The MRI examinations were read by an experienced radiologist (SYH) with 5 years of experience in pelvic MRI, blinded for the final histological diagnosis.

### FDG-PET/CT and derived imaging parameters

PET/CT was performed on a Biograph 40 True Point scanner (Siemens). The scanning covered from the caput to the proximal thigh. The protocol included 6 h of fasting prior to imaging. 18F-FDG (322–414 MBq) was given intravenously 60–120 min prior to scanning. Low-dose CT (120 kV, 50 mAs) for attenuation correction of the PET data was acquired before the static emissions, which were obtained at intervals of 3 min per bed position; subsequently, intravenous contrast agent (Iomerol, 350 mg iodine/mL; Bracco Imaging Scandinavia, AB) and negative oral contrast agent (water) were administered for the diagnostic CT scan (120 mV, 240 mAs). The PET images were fused with both the diagnostic and the low-dose CT images and the metabolic tumor measurements were performed using the low-dose fusion images. Mean/median (range) interval between PET-scanning and surgery was 19/13 (0-283) days. The two patients with longest interval (283 and 174 days) between scanning and primary surgery had had conservative treatment prior to surgery. The PET-CT examinations were read by a nuclear medicine physician (AG) with three years of experience in nuclear medicine, blinded for the final histological diagnosis. The tumor SUV_max_ was recorded and metabolic tumor volume (MTV) and SUV_mean_ were measured in a volume of interest (VOI) including voxels with an SUV of more than 2.5. TLG in the tumor was also estimated using the following equation: TLG = SUV_mean_ × MTV [37].

### Oligonucleotide DNA microarray analyses

RNA was extracted from fresh frozen tumor tissue in the area of highest tumor purity. The bulk of samples had tumor content above 80%, and the minimum inclusion threshold was set to >50%. The RNA was hybridized to Agilent Whole Human Genome Microarrays according to instruction from the vendor (www.agilent.com). The arrays were then scanned by the Agilent Microarray Scanner Bundle. Intensity of the spot signal was interpreted by using the software J-Express (http://jexpress.bioinfo.no/site/).

Samples diagnosed by pathologist as CAH or EECG1 were selected, and unsupervised hierarchical clustering was conducted using Euclidean distance measurement. Transcriptional differences between two groups were explored by GSEA [17], applying pre-defined gene sets supplied by the MSigDB (www.broadinstitute.org/gsea/index.jsp). SAM was used to identify genes most differentially expressed in two groups of patients.

### Immunohistochemical staining

Tissue micro arrays (TMAs) were prepared as previously described, selecting three 0.6 mm cylinders from the most representative tumor area on formalin fixed paraffin embedded (FFPE) tissue blocks from all patients [38]. Immunohistochemical staining of the proteins PBK and NEK2 were conducted using the following procedure: Dewaxing of the slides in xylene was followed by rehydration in ethanol. Subsequently the slides were boiled for 20 min in Target retrieval buffer pH 9, followed by application of peroxidase block for 5 min. Slides were incubated with the primary antibody (PBK diluted 1:50, NEK2 diluted 1:500) (PBK: Cell Signaling, #4942, NEK2: Abcam, ab55550) at room temperature for 60 min. Secondary antibody (Dako, EnVision and labelled anti-rabbit for PBK and anti-mouse for NEK2) were applied for 30 min followed by DAB^+^ before counterstained with Hematoxylin.

The stained slides were evaluated using the well-established semi-quantitative staining index graded from 0-9, being a product of staining intensity (0-3) and the area with this intensity (0-3) [38]. PBK protein expression was mainly nuclear and found in the epithelial tumor component. Cut-off values for high PBK protein expression were defined as upper tertile, and low expression as the lower two tertiles, based on the number of events and size of the patient groups in a survival analysis. For NEK2 no cut-off was determined as unspecific staining resulted in high staining index for all lesions.

### Statistical analysis

The statistical software SPSS (Statistical Package of Social Science) version 23.0 was used for analyses. All reported p-values were two-sided, and values under 0.05 were regarded as significant. Differences in continuous data were assessed by non-parametric Mann-Whitney-U test. Pearson-Chi-squared test and Fisher's exact test were used to investigate differences in categorical data. ROC curves were generated to evaluate the diagnostic value of the different imaging markers in discriminating CAH from EECG1. The optimal cut-off values (rounded to one decimal) were determined for which the best separation in Youden index between groups was achieved. Non-parametric Mann-Whitney-U test was used to validate the significance of the cut-off. Disease specific survival was analyzed using Kaplan-Meier method (log rank test), with date of primary surgical treatment as entry date and the date of death due to endometrial cancer as endpoint.

## SUPPLEMENTARY MATERIALS FIGURES AND TABLES



## Abbreviations

CAH: complex atypical hyperplasia
BMI: body mass index
MRI: magnetic resonance imaging
FDG-PET/CT: fluorodeoxyglucose positron emission tomography/computer tomography
EECG1: endometrioid endometrial cancers grade 1
SUV_max_: maximum standardized uptake value
SUV_mean_: mean standardized uptake value
TLG: total lesion glycolysis
ROC: receiver operating characteristic
SAM: significance analysis of microarrays
GSEA: gene set enrichment analysis
EMT: epithelial to mesenchymal transition
PBK: PDZ-binding kinase
NEK2: never in mitosis related kinase 2
MELK: maternal embryonic leucine zipper kinase
CCNB1: cyclin B1
IHC: immunohistochemistry
FIGO: International federation of gynecology and obstetrics
PR: progesterone receptor
EIN: endometrial intraepithelial neoplasia
ER: estrogen receptor
PTEN: phosphatase and tensin homolog
CIN: cervical intraepithelial neoplasia
MTV: metabolic tumor volume
TMA: tissue micro arrays
FFPE: formalin fixed paraffin embedded.
