# Supplementary material for: Preoperative imaging markers and PDZ-binding kinase tissue expression predict low-risk disease in endometrial hyperplasias and low grade cancers
Source: Oncotarget. 2017 Jul 31;8(40):68530–41. doi: 10.18632/oncotarget.19708 (PMC5620275; doi:10.18632/oncotarget.19708)
Supplement: Supplementary file 1 [file oncotarget-08-68530-s001.pdf]

Preoperative imaging markers and PDZ-binding kinase tissue expression predict low-risk disease in endometrial hyperplasias and low grade cancers

SUPPLEMENTARY MATERIALS

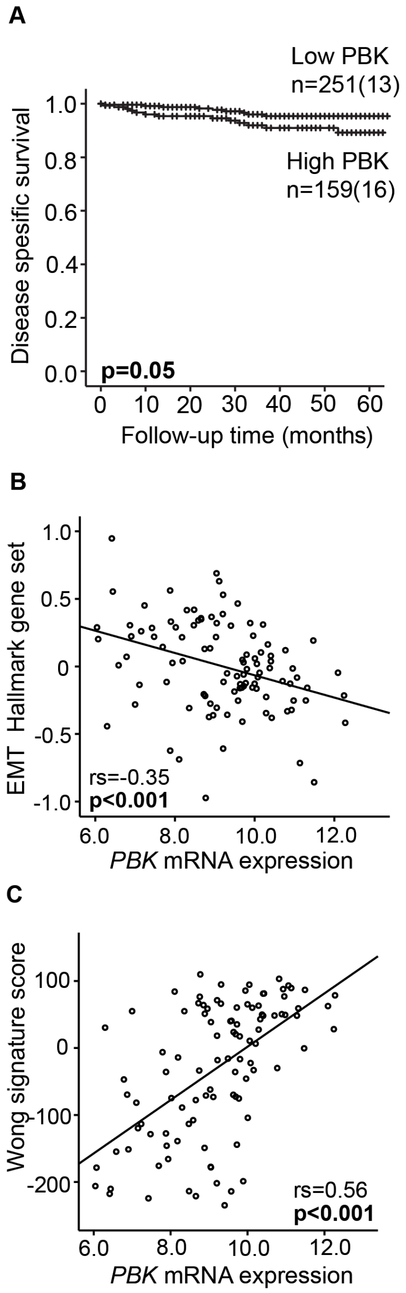

Supplementary Figure 1: PBK in relation to survival and relevant gene signatures. (A) Endometrioid endometrial cancer (EEC) patients with high PBK protein expression had reduced survival (n=410). **(B-C)** Gene set enrichment analysis (GSEA) conducted in 102 patients with complex atypical hyperplasia (CAH) to endometrioid endometrial cancer grade 1 (EECG1). EMT Hallmark gene set was significantly anti-correlated *PBK* mRNA expression. Wong signature score (base on curated gene sets) was significantly correlated to *PBK* mRNA expression.

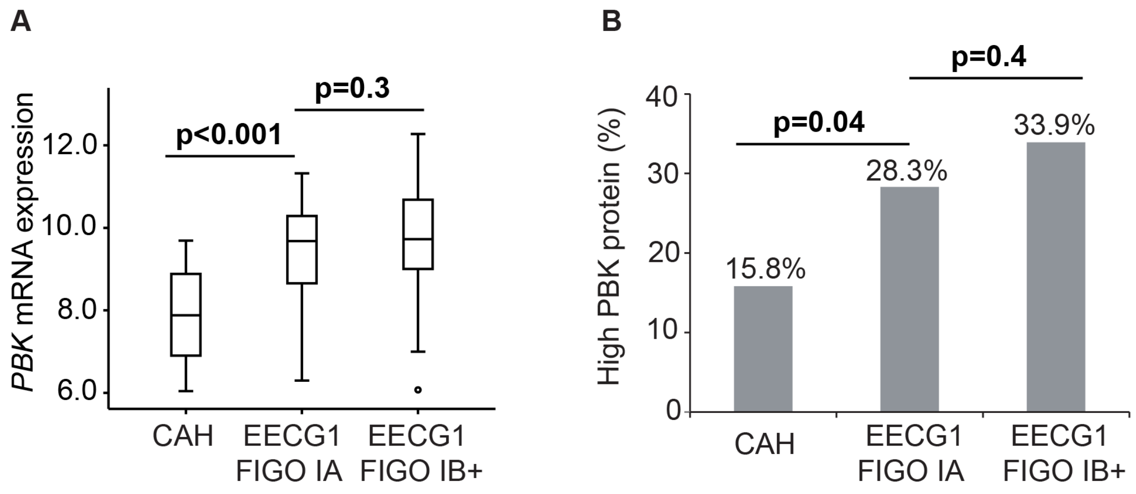

Supplementary Figure 2: PBK expression according to histological type and disease extent. (A) *PBK* mRNA expression increase from complex atypical hyperplasia (CAH) to endometrioid endometrial cancer grade 1 (EECG1) FIGO stage 1A, but not further to higher FIGO stage. **(B)** High protein expression of PBK is more frequent in EECG1 stage 1A than CAH lesions, however, not in higher FIGO stage.

Supplementary Table 1: Gene set enrichment analysis (GSEA) conducted in 102 patients with CAH or EECG1

Top-ranked Hallmark gene sets up-regulated in Cluster I				
**Rank**	**Gene set**	**Size**	**p-value**	**FDR**
**1**	HALLMARK_EPITHELIAL_MESENCHYMAL_TRANSITION	194	0.00	0.0
**2**	HALLMARK_UV_RESPONSE_DN	136	0.00	0.0
**3**	HALLMARK_MYOGENESIS	195	0.00	0.0
**4**	HALLMARK_HEDGEHOG_SIGNALING	36	0.01	0.49
**5**	HALLMARK_COAGULATION	138	0.00	0.55
**6**	HALLMARK_APICAL_JUNCTION	192	0.00	0.52
**7**	HALLMARK_TGF_BETA_SIGNALING	51	0.02	1.14
**8**	HALLMARK_KRAS_SIGNALING_UP	192	0.00	1.84
**9**	HALLMARK_WNT_BETA_CATENIN_SIGNALING	40	0.03	2.14
**10**	HALLMARK_NOTCH_SIGNALING	30	0.04	2.55
**Top-ranked Hallmark gene sets up-regulated in Cluster II**				
**Rank**	**Gene set**	**Size**	**p-value**	**FDR**
**1**	HALLMARK_E2F_TARGETS	177	0.00	0.0
**2**	HALLMARK_G2M_CHECKPOINT	181	0.00	0.0
**3**	HALLMARK_MYC_TARGETS_V1	178	0.00	0.0
**4**	HALLMARK_MTORC1_SIGNALING	192	0.00	0.0
**5**	HALLMARK_OXIDATIVE_PHOSPHORYLATION	195	0.00	0.0
**6**	HALLMARK_GLYCOLYSIS	190	0.00	0.0
**7**	HALLMARK_MYC_TARGETS_V2	50	0.00	0.0
**8**	HALLMARK_UNFOLDED_PROTEIN_RESPONSE	109	0.00	0.0
**9**	HALLMARK_FATTY_ACID_METABOLISM	154	0.00	0.0
**10**	HALLMARK_REACTIVE_OXIGEN_SPECIES_PATHWAY	47	0.00	0.0

Supplementary Table 2: Gene set enrichment analysis (GSEA) conducted in 102 patients with CAH or EECG1

Top-ranked curated gene sets up-regulated in Cluster I				
**Rank**	**Gene set**	**Size**	**p-value**	**FDR**
**1**	WONG_ENDMETRIUM_CANCER_DN	74	0.00	0.0
**2**	LIM_MAMMARY_STEM_CELL_UP	452	0.00	0.0
**3**	BOQUEST_STEM_CELL_UP	243	0.00	0.0
**4**	SCHUETZ_BREAST_CANCER_DUCTAL_INVASIVE_UP	330	0.00	0.0
**5**	LIU_PROSTATE_CANCER_DN	422	0.00	0.0
**6**	SMID_BREAST_CANCER_NORMAL_LIKE_UP	430	0.00	0.0
**7**	ANASTASSIOU_CANCER_MESENCHYMAL_ TRANSITION_SIGNATURE	62	0.00	0.0
**8**	ONDER_CDH1_TARGETS_2_UP	230	0.00	0.0
**9**	NABA_CORE_MATRISOME	244	0.00	0.0
**10**	LINDGREN_BLADDER_CANCER_CLUSTER_2B	366	0.00	0.0
**Top-ranked curated gene sets up-regulated in Cluster II**				
**Rank**	**Gene set**	**Size**	**p-value**	**FDR**
**1**	ROSTY_CERVICAL_CANCER_PROLIFERATION_ CLUSTER	129	0.00	0.0
**2**	SOTIRIOU_BREAST_CANCER_GRADE_1_VS_3_UP	138	0.00	0.0
**3**	KOBAYASHI_EGFR_SIGNALING_24HR_DN	230	0.00	0.0
**4**	SHEDDEN_LUNG_CANCER_POOR_SURVIVAL_A6	411	0.00	0.0
**5**	DUTERTRE_ESTRADIOL_RESPONSE_24HR_UP	294	0.00	0.0
**6**	BENPORATH_PROLIFERATION	135	0.00	0.0
**7**	VECCHI_GASTRIC_CANCER_EARLY_UP	373	0.00	0.0
**8**	CHANG_CYCLING_GENES	132	0.00	0.0
**9**	ZHOU_CELL_CYCLE_GENES_IN_IR_RESPONSE_24HR	115	0.00	0.0
**10**	WHITEFORD_PEDIATRIC_CANCER_MARKERS	108	0.00	0.0

Supplementary Table 3: Top-ranked genes differentially expressed between Cluster I and Cluster II (FDR=0) and the highest fold change

Most differentially up-regulated genes in Cluster II.		Most differentially down-regulated genes in Cluster II.	
**Gene name**	**Fold change**	**Gene name**	**Fold change**
LEFTY1	4.92	TNXB	8.08
CA9	4.53	MGC16121	8.01
CTSL2	4.12	NBLA00301	7.71
UPK1B	4.11	TRH	7.52
MMP12	3.87	FOXL2	7.07
PBK	3.82	A_32_P171043	7.05
NMU	3.73	AK125162	6.89
C19orf21	3.58	CXCL12	6.48
OIP5	3.53	ENST00000334770	6.12
CCNB2	3.52	DPP6	5.88
E2F7	3.45	OSR2	5.87
CEACAM6	3.43	OSR2	5.86
TFF3	3.42	OGN	5.61
KIAA0101	3.41	SFRP4	5.53
TFF3	3.39	MEG3	5.46
PCSK9	3.33	AA837799	5.41
MAL	3.3	TSHZ3	5.37
NEK2	3.29	MFAP4	5.32
MELK	3.28	TWIST2	5.32
MESP1	3.27	RP13-347D8.3	5.3
CKMT1A	3.26	LOC730125	5.27
LCN2	3.24	WIT1	5.27
RHOV	3.23	HAND2	5.25
DLG7	3.22	F10	4.94
CCNB1	3.21	CLEC3B	4.82

Genes yielding best separation between clusters are highlighted in yellow.

Supplementary Table 4: Preoperative quantitative imaging parameter for the endometrial lesions belonging to Cluster I versus Cluster II in 53 patients among whom the final diagnosis was complex atypical hyperplasia (CAH) (n=5) and endometrioid endometrial cancer grade 1 (EECG1) (n=48) based on hysterectomy

	Cluster I Mean (n)	Cluster II Mean (n)	p-value*
**MRI**			
ADC ($\times 10^{-6}$ mm^2/s)	928 (19)	766 (30)	**0.001**
Volume (ml)	6.6 (22)	19.5 (31)	**0.03**
**FDG-PET-CT**			
SUV_{max}	10.4 (8)	16.3 (17)	**0.03**
SUV_{mean}	4.1 (8)	6.4 (17)	**0.001**
MTV (ml)	11.7 (8)	30.8 (17)	0.12
TLG (g)	53.6 (8)	234.8 (17)	**0.02**

*Mann Whitney U test.
ADC, apparent diffusion coefficient; FDG PET-CT, fluorodeoxyglucose positron emission tomography/computer tomography; MRI, magnetic resonance imaging; MTV, metabolic tumor volume; SUV_{max}, maximum standard uptake value; SUV_{mean}, mean standard uptake value; TLG, total lesion glycolysis. Significant p-values are given in **bold**.

Supplementary Table 5: Preoperative quantitative imaging parameters and corresponding PBK protein expression levels (high/low) in endometrial lesions of 94 patients diagnosed with complex atypical hyperplasia (CAH) (n=13) and endometrioid endometrial cancer grade 1 (EECG1) (n=81)

Quantitative imaging parameter	PBK protein expression		p-value*
	Low	High	
	Mean (n)	Mean (n)	
**MRI**			
ADC ($\times 10^{-6}$ mm^2/s)	874 (61)	813 (26)	0.07
Volume (ml)	8.3 (65)	16.1 (29)	**0.03**
**FDG-PET-CT**			
SUV_{max}	11.0 (32)	15.8 (21)	**0.02**
SUV_{mean}	4.8 (30)	6.1 (21)	**0.03**
MTV (ml)	20.1 (30)	26.7 (21)	0.25
TLG (g)	121.5 (30)	199.8 (21)	0.10

*Mann Whitney U test.

ADC, apparent diffusion coefficient; FDG-PET/CT, fluorodeoxyglucose positron emission tomography; MRI, magnetic resonance imaging; MTV, metabolic tumor volume; SUV_{max}, maximum standard uptake value; SUV_{mean}, mean standard uptake value; TLG, total lesion glycolysis. Significant p-values are given in **bold**.
